# Asthma and Summary Measure of Social Determinants of Health/Health Equity Among Adults in Mississippi, 2022

**DOI:** 10.5888/pcd22.240444

**Published:** 2025-09-18

**Authors:** Vincent L. Mendy, Tawandra L. Rowell-Cunsolo, Byambaa Enkhmaa

**Affiliations:** 1 Department of Epidemiology and Biostatistics, College of Health Sciences, Jackson State University, Jackson, Mississippi; 2 Sandra Rosenbaum School of Social Work, University of Wisconsin-Madison; 3 Department of Internal Medicine, School of Medicine, University of California, Davis

## Abstract

Social determinants of health (SDOH) contribute to asthma prevalence and disparities in health outcomes. We used data from 3,994 respondents to the Social Determinants and Health Equity (SD/HE) module of the 2022 Mississippi Behavioral Risk Factor Surveillance System survey to examine the association between a summary measure of SDOH and the prevalence of self-reported asthma among adults in Mississippi. Adults experiencing 1 (adjusted odds ratio [AOR], 1.67), 2 (AOR, 1.69) or 4 or more (AOR, 2.33) SD/HE risk factors had higher odds of asthma compared with those experiencing no SD/HE risk factors. Our findings suggest a need to develop interventions for adults in Mississippi with multiple SDOH/HE risk factors.

**Summary**

**What is already known on this topic?**

Social determinants of health contribute to excess risk of asthma.

**What is added by this report?**

Adults in Mississippi who reported experiencing 1, 2, or 4 or more social determinants of health and health equity (SDOH/HE) risk factors had significantly higher odds of asthma than those experiencing no SDOH/HE risk factors.

**What are the implications for public health practice?**

Interventions and screenings aimed at reducing SDOH/HE risk factors may help reduce the prevalence of asthma among adults in Mississippi.

## Objective

Asthma is a chronic disease associated with high morbidity and mortality among adults (1). Among adults, asthma may accelerate the decline of lung function (1), a risk factor for lung disease and mortality (2). Asthma is also associated with cardiovascular disease and all-cause mortality (3). The prevalence of asthma among adults in Mississippi increased significantly from 12.5% in 2013 to 15.1% in 2022 (4).

Social determinants of health (SDOH), or social conditions such as housing, transportation, neighborhood characteristics, access to nutritious foods, experience(s) with discrimination, and health care access, have a major effect on health, well-being, and quality of life (5,6). SDOH contribute to the excess prevalence of asthma, asthma-related morbidity, and abnormal lung function among some populations (2). Because SDOH risk factors or experiences are highly interrelated, a composite measure of SDOH could provide a more comprehensive picture of the causes of disparities in health outcomes than an approach that assesses a single social risk factor (7). Although research has demonstrated associations between social conditions and asthma, data on summary measures of SDOH and health equity (SDOH/HE) and asthma in Mississippi are limited. We examined the associations between asthma prevalence and a summary measure of SDOH/HE among adults in Mississippi.

## Methods

We analyzed data from the 2022 Mississippi Behavioral Risk Factor Surveillance System (BRFSS) survey, including the Social Determinants and Health Equity (SD/HE) module, newly introduced in that year. Detailed information about BRFSS is available elsewhere (www.cdc.gov/brfss). We restricted analyses to respondents (n = 3,994) who self-identified as either non-Hispanic Black or non-Hispanic White. This study was deemed exempt by the Jackson State University institutional review board.

We defined asthma as a yes response to the following question: “Has a doctor, nurse, or other health professional ever told you that you had asthma?” Answers were coded as yes or no.

In 2022, 39 states, the District of Columbia, and 2 US territories (Puerto Rico and the US Virgin Islands) collected SD/HE data. We used responses to 10 questions from this module (Table 1). These questions, based on the Center for Medicare and Medicaid Innovation’s Health-Related Social Needs Screening Tool (7), asked about employment/economic stability, receipt of food stamps or the Supplemental Nutrition Assistance Program, housing stability and quality, food security, transportation access, utilities security, social isolation, social and emotional support, life satisfaction, and mental well-being (7). To create an SDOH/HE summary measure, we categorized the responses to each SD/HE item as 0 (minimal or no experience) or 1 (experience) and then summed all scores. The resulting summary SDOH/HE scores ranged from 0 to 10; we then categorized respondents as having 0, 1, 2, 3, or 4 or more risk factors (7).

**Table 1:** The Social Determinants and Health Equity (SD/HE), Behavioral Risk Factor Surveillance System, 2022

Doman	Question	Answer
1. Employment/economic stability	“In the past 12 months have you lost employment or had hours reduced?”	1 Yes 2 No 7 Don’t know/not sure 9 Refused
2. Receiving food stamps or Supplemental Nutrition Assistance Program (SNAP)	“During the past 12 months, have you received food stamps, also called SNAP, the Supplemental Nutrition Assistance Program on an EBT card?”	1 Yes 2 No 7 Don’t know/not sure 9 Refused
3. Housing stability and quality	“During the last 12 months, was there a time when you were not able to pay your mortgage, rent or utility bills?”	1 Yes 2 No 7 Don’t know/not sure 9 Refused
4. Food security	“During the past 12 months how often did the food that you bought not last, and you didn't have money to get more?”	1 Always 2 Usually 3 Sometimes 4 Rarely 5 Never 7 Don’t know/not sure 9 Refused
5. Transportation access	“During the past 12 months has a lack of reliable transportation kept you from medical appointments, meetings, work, or from getting things needed for daily living?”	1 Yes 2 No 7 Don’t know/not sure 9 Refused
6. Utilities security	“During the last 12 months was there a time when an electric, gas, oil, or water company threatened to shut off services?”	1 Yes 2 No 7 Don’t know/not sure 9 Refused
7. Social isolation	“How often do you feel socially isolated from others?”	1 Always 2 Usually 3 Sometimes 4 Rarely 5 Never 7 Don’t know/not sure 9 Refused
8. Social and emotional support	“How often do you get the social and emotional support that you need?”	1 Always 2 Usually 3 Sometimes 4 Rarely 5 Never 7 Don’t know/not sure 9 Refused
9. Life satisfaction	“In general, how satisfied are you with your life?”	1 Very satisfied 2 Satisfied 3 Dissatisfied 4 Very dissatisfied 7 Don’t know/not sure 9 Refused
10. Mental well-being	“Stress means a situation in which a person feels tense, restless, nervous or anxious or is unable to sleep at night because their mind is troubled all the time. Within the last 30 days, how often have you felt this kind of stress?”	1 Always 2 Usually 3 Sometimes 4 Rarely 5 Never 7 Don’t know/not sure 9 Refused

We used a logistic regression model to estimate adjusted odds ratios (AORs) and 95% CIs for the association between the prevalence of asthma and the summary measure of SDOH/HE. The logistic regression model included age, race, sex, education, income, smoking, exercise, health insurance, body mass index, and diabetes status. We used SAS version 9.4 (SAS Institute, Inc) to perform all statistical analyses, accounting for the complex sample design.

## Results

The mean age of respondents was 49.0 years. More than one-third (37.6%) were non-Hispanic Black, more than half (53.7%) were women, and 44.0% had an annual household income of $50,000 or more. Asthma prevalence was 14.7%, and the likelihood of experiencing 0, 1, 2, 3, or 4 or more SDOH/HE risk factors was 38.9% (95% CI, 36.7%–41.0%), 21.9% (95% CI, 20.0%–23.8%), 13.8% (95% CI, 12.4%–15.3%), 8.5% (95% CI, 7.3%–9.7%), and 16.9% (95% CI, 15.3%–18.6%), respectively.

Adults in Mississippi experiencing 1 risk factor (AOR, 1.67; 95% CI, 1.10–2.55), 2 risk factors (AOR, 1.69; 95% CI, 1.07–2.65), or 4 or more risk factors (AOR, 2.33; 95% CI, 1.47–3.69) had higher odds of asthma compared with those experiencing no risk factors (Table 2).

**Table 2:** Association Between Asthma and a Summary Measure of SDOH/HE Among Adults in Mississippi, Behavioral Risk Factor Surveillance System, 2022

No. of SDOH/HE risk factors	Unadjusted OR (95% CI)	Adjusted OR^a^ (95% CI)
0	1 [Reference]	1 [Reference]
1	1.64 (1.15–2.34)	1.67 (1.10–2.55)
2	1.90 (1.29–2.79)	1.69 (1.07–2.65)
3	1.60 (0.95–2.68)	1.20 (0.67–2.15)
≥4	2.71 (1.94–3.77)	2.33 (1.47–3.69)

Abbreviations: OR, odds ratio; SDOH/HE, social determinants of health/health equity.

a Adjusted for age, race, sex education, income, smoking, exercise, health insurance, body mass index, and diabetes status.

## Discussion

Our results showed that presence of 1, 2, or 4 or more SDOH/HE risk factors was associated with an increased prevalence of asthma among adults in Mississippi. These findings are consistent with a recent national study of adults in 39 states, the District of Columbia, and 2 US territories, which assessed 12 indicators of SDOH and health-related social needs (SDOH/HRSN) and indicated that adults with asthma were more likely than adults without asthma to have more than 3 adverse SDOH/HRSN indicators (8). Additionally, the study reported that, generally, adults with chronic health conditions were more likely than adults without those conditions to have SDOH/HRSN risk factors. Our earlier research demonstrated that adults in Mississippi with 4 or more SDOH/HE risk factors had significantly higher odds of cardiovascular disease than those with no SDOH/HE risk factors (9). Finally, our current results are well-aligned with observational and epidemiologic research suggesting that SDOH, including socioeconomic status and housing, contribute to an excess burden of asthma (2).

Our findings demonstrate the importance of using a summary measure of SDOH/HE when examining the prevalence of chronic disease. Given substantial evidence that SDOH negatively influence the prevalence and severity of asthma, public health practitioners could use a composite measure of SDOH/HE to inform screening strategies and develop targeted interventions to reduce the disproportionate prevalence of asthma in the state. The lack of association between the presence of 3 SDOH/HE risk factors and asthma could be due to the low percentage of participants (8.5%) who had 3 risk factors; this finding warrants further investigation.

Improved SDOH measurement is important in addressing gaps in health equity (10). Expanding the use of SDOH/HE measures to assess the relationships between SDOH and chronic disease prevalence may help in developing targeted interventions to address persistent health inequities among medically and socioeconomically marginalized populations.

This analysis has several limitations. BRFSS data are self-reported and thus are subject to recall and social desirability bias (11). A summary measure of SDOH/HE may mask individual effect or potential interactions between factors. The SD/HE module was limited to certain domains. Associations were cross-sectional and thus did not permit causal inferences.

Interventions and screenings, such as those described in the Protocol for Responding to and Assessing Patients’ Assets, Risks and Experiences (PRAPARE) (12) may help reduce the prevalence of asthma among adults in Mississippi.
